# The Future of Imaging in Heart Failure: Toward Precision Phenotyping, Integration, and Intelligence

**DOI:** 10.1007/s11897-026-00769-6

**Published:** 2026-07-22

**Authors:** Moritz J Hundertmark

**Affiliations:** Department of Cardiology, University Hospital Inselspital Bern, Bern, Switzerland

**Keywords:** Heart failure, Multimodality imaging, Artificial intelligence, Cardiovascular magnetic resonance, Molecular imaging, Digital twins

## Abstract

**Purpose of Review:**

Heart failure (HF) is increasingly understood not as a single, uniformly treated diagnosis but as a heterogeneous syndrome requiring aetiological clarification, in which cardiac imaging is central. As the opening article of this journal's *‘Imaging in Heart Failure’* section, this review surveys the technologies currently reshaping HF imaging and sets out the section's scope and priorities, framing the shift from a descriptive, modality-siloed practice toward an integrated, predictive, patient-specific discipline.

**Recent Findings:**

Artificial intelligence (AI) now delivers expert-level echocardiography automation, guides image acquisition by novices in resource-limited settings, detects aetiologies such as transthyretin amyloid cardiomyopathy from a single acquisition and enables deep phenotyping through radiomics and vendor-agnostic strain analysis. Handheld, AI-enabled point-of-care ultrasound extends imaging-guided triage beyond the echocardiography laboratory. Cardiovascular magnetic resonance (CMR) advances — parametric mapping, four-dimensional flow, diffusion tensor imaging, spectroscopy, and accelerated reconstruction — broaden tissue and metabolic characterisation, including patients with implanted devices. Molecular imaging with novel positron emission tomography tracers and hyperpolarised magnetic resonance is moving from depicting the structural consequences of disease to imaging active pathobiology, while photon-counting computed tomography and image-derived digital twins support one-stop structural assessment and in-silico prediction of therapy response.

**Summary:**

The convergence of AI, molecular imaging and advanced precision is transforming HF imaging from better pictures into smarter, integrated, personalised data that directly inform care. Realising this promise will require rigorous validation, attention to algorithmic bias and generalisability, demonstrated cost-effectiveness, curricular reform, and equitable access. This section aims to critically appraise these innovations and their translation into practice.

Heart failure (HF) has long been considered an isolated problem of western, high-income nations with an aging population. Recently however, its prevalence has soared in lower- and middle-income countries, leading to more than 60 million affected individuals globally [1]. A substantial contributor to this increasing prevalence is improved diagnostics via dedicated imaging modalities, offering indubitable diagnoses. Current imaging approaches have transformed our understanding of HF from being a final diagnosis (‘what’) treated uniformly, to a question in need of further investigation to find the underlying cause (‘why’). Echocardiography remains the easiest accessible first-line modality, cardiac magnetic resonance (CMR) provides a ‘one-stop-shop’ approach including accurate assessment of myocardial volumes, function and tissue characterisation, while nuclear imaging offers functional and metabolic insights and cardiac computed tomography (CCT) increasingly contributes to structural and coronary assessment [2]. Fuelled by advances in artificial intelligence (AI), the field of cardiac imaging is undergoing a profound transformation promising multimodality integration, molecular and metabolic imaging across modalities, next-generation computational modelling and the emergence of digital twins. Those developments are poised to transition cardiac imaging from a descriptive, modality-siloed discipline into an integrated, predictive, and patient-specific science [3] (see Fig. 1). 

**Fig. 1:**
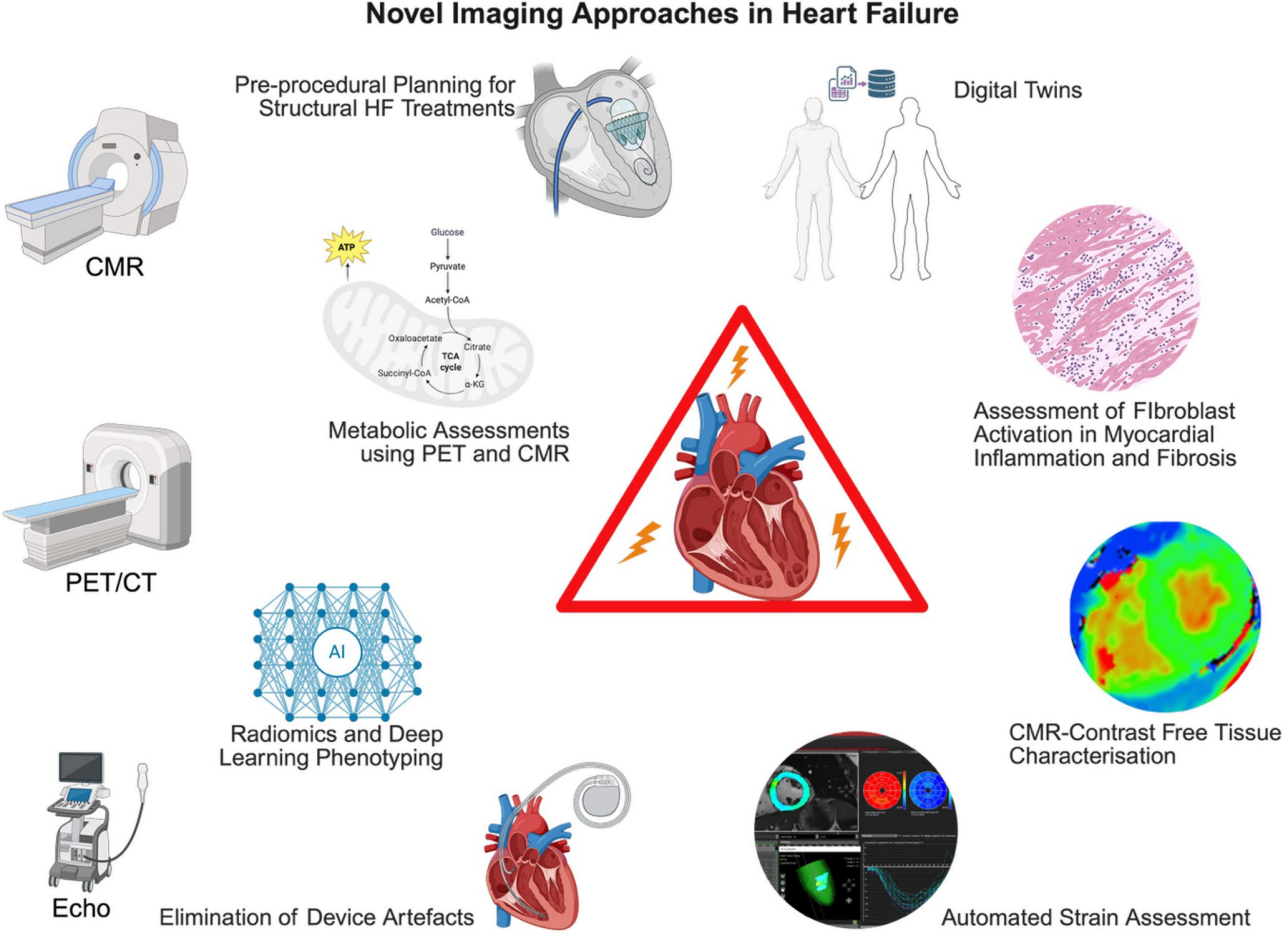
Novel imaging approaches improving diagnosis, risk stratification and prognosis in patients with HF. Echo= echocardiography, CMR= cardiovascular magnetic resonance, CT= computer tomography, PET=positron emission tomography. Figure created with BioRender using privately owned images

## Artificial Intelligence and Deep Learning in HF Imaging

Despite the breadth of available imaging data, clinical integration remains fragmented, inter-observer variability persists, and heterogeneity in HF phenotypes often escapes conventional imaging approaches.

The routine integration of AI into cardiac imaging represents perhaps the most impactful near-term transformation. Deep learning algorithms have demonstrated expert-level performance in automated echocardiographic view classification, chamber quantification, and ejection fraction estimation, with large-scale validation studies showing that AI-derived measurements are at least as reproducible as human expert analysis [4]. Implementation of a deep-learning AI algorithm is equally useful to train novice human sonographers in acquiring diagnostic echocardiographic images, an observation especially impactful for resource constrained settings, such as lower income regions [5]. Additionally, different approaches now enable automatic identification of underlying HF-aetiologies, such as transthyretin amyloidosis cardiomyopathy (ATTR-CM) from a single echocardiographic image acquisition [6, 7] while clustering algorithms may improve classification of distinct phenotypes which bears prognostic relevance [8].

AI-enabled *deep phenotyping*—the extraction of imaging features invisible to the human eye— represents another field of interest. Radiomics and texture analysis applied to CMR images can predict outcomes in dilated cardiomyopathy and identify sub-phenotypes within the heterogeneous HF-populations, which in turn has implications for outcomes [9, 10]. Foundation models, trained on large multimodal datasets, are beginning to integrate imaging with electronic health record data, creating holistic patient representations that may enable individual risk stratification and therapy.

A critical frontier is the application of AI to strain imaging. Myocardial deformation analysis by speckle-tracking echocardiography (STE) and CMR feature tracking has well-established prognostic value in HF, but its clinical adoption has been limited by vendor dependency and analysis complexity. Vendor-agnostic, AI-powered strain analysis promises to democratise access to this powerful biomarker and enable its integration into routine clinical workflows [11].

## Next-generation Echocardiography

Echocardiography continues to evolve at a rapid pace. Three-dimensional (3D) echocardiography provides volumetric assessments that more closely approximate CMR-derived reference standards, and improvements in temporal resolution and automated border detection are expanding its practical applicability in HF clinics. Point-of-care ultrasound (POCUS), facilitated by handheld devices with on-device AI, is another significant development. These devices can screen for reduced ejection fraction, elevated filling pressures, and pleural effusions with meaningful diagnostic accuracy, enabling imaging-guided HF triage in community, emergency, and resource-limited settings [12]. The combination of POCUS with wearable biosensors and remote monitoring platforms creates the possibility of a continuous, imaging-augmented HF surveillance ecosystem.

## Advances in Cardiac Magnetic Resonance

CMR remains the gold standard for myocardial tissue characterisation and is central to the aetiological evaluation of newly diagnosed HF. Parametric mapping techniques (T1, T2, T2*, and extracellular volume [ECV] mapping) have matured considerably, enabling quantitative assessment of myocardial fibrosis, oedema, and iron overload without the need for gadolinium-based contrast agents in many scenarios.

Emerging CMR techniques with relevance to HF include four-dimensional (4D) flow imaging, which provides comprehensive assessment of intracardiac flow patterns and energetics, diffusion tensor imaging (DTI) for the non-invasive evaluation of myocardial microstructure and fibre architecture, and magnetic resonance spectroscopy (MRS) for metabolic profiling of the failing myocardium [13]. Accelerated acquisition strategies, including compressed sensing and AI-based reconstruction, are reducing scan times substantially, making CMR more practical for serial monitoring in HF patients, including those with implanted electronic devices [14].

## Molecular and Nuclear Imaging

Molecular imaging offers a unique window into the pathobiology of HF at the cellular and molecular level. Positron emission tomography (PET) with novel tracers is advancing beyond myocardial perfusion and viability assessment into the domains of cardiac inflammation, sympathetic innervation, and metabolic remodelling. ^18^F-fluorodeoxyglucose (^18^F-FDG) PET is increasingly used to detect active cardiac sarcoidosis and myocarditis, while ^123^I-metaiodobenzylguanidine (MIBG) imaging provides prognostic information through assessment of cardiac sympathetic denervation [15].

Newer PET tracers targeting fibroblast activation protein (FAP) are generating considerable excitement in the HF community. FAP is upregulated in activated cardiac fibroblasts, and ^68^Ga-labelled FAP inhibitor (FAPI) PET has shown the ability to detect active fibrosis in myocardial infarction, myocarditis, and various cardiomyopathies, potentially offering an imaging biomarker for a process that was previously assessable only by biopsy [14]. This represents a conceptual leap from imaging structural consequences of fibrosis (by CMR) to imaging the active fibrotic process itself.

Hyperpolarized MR, a cutting-edge technique designed to enhance sensitivity to MR by a factor of 10.000, enables visualisation and quantification of metabolic disturbances of the heart in select HF populations, representing an in-vivo approach for comprehensive HF phenotyping and assessment of novel drugs [16].

## Cardiac Computed Tomography and Structural Assessment

The role of CCT in HF is expanding beyond coronary artery assessment. CCT-derived fractional flow reserve (FFR-CT) enables non-invasive functional assessment of coronary lesions in patients with ischaemic HF. Furthermore, advanced acquisition and post-processing techniques harnessing photon-counting CT (PCCT), including CT-derived strain, ECV quantification, and myocardial perfusion analysis, are establishing CCT as a potential one-stop-shop for certain HF patients [17]. In the rapidly growing field of structural heart interventions, including transcatheter mitral and tricuspid valve therapies for functional regurgitation in HF, CCT provides essential preprocedural planning through detailed anatomical characterisation.

## Digital Twins and Computational Modelling

The concept of the cardiac digital twin—a personalised, image-derived computational model of an individual patient’s heart—represents a transformative frontier in HF imaging. These models integrate imaging data (typically from CMR or CCT) with electrophysiological and haemodynamic information to create patient-specific simulations that can predict responses to therapies in silico before they are applied clinically [18].

In HF, digital twin technology holds particular promise for optimising cardiac resynchronisation therapy (CRT) by predicting optimal lead positions and programming parameters, guiding surgical ventricular restoration, and modelling the haemodynamic impact of device therapies such as left ventricular assist devices (LVADs) [19]. Although still largely in the research domain, several EU- and US-funded consortia are actively working toward clinical translation, and regulatory frameworks for in silico clinical trials using digital twins are being developed.

## Challenges and the Translational Roadmap

Despite the remarkable pace of innovation, significant challenges remain. The validation and regulatory approval of AI algorithms require large, diverse, and rigorously annotated datasets, and issues of algorithmic bias, generalisability, and interpretability must be addressed before widespread clinical deployment. The cost-effectiveness of advanced imaging strategies in HF remains to be demonstrated in prospective outcomes studies. More importantly, physician training must evolve to equip the next generation of HF-specialists with competencies in AI, computational imaging, and multimodality integration.

The equitable dissemination of advanced imaging technologies remains a pressing concern. Many innovations discussed in this review are currently available only in well-resourced academic centres, and deliberate strategies are needed to ensure that advances in HF imaging benefit all patients, regardless of geography or socioeconomic status. Handheld AI-enabled ultrasound devices and cloud-based analysis platforms represent promising avenues for democratising access.

## Conclusions and Vision for this Section

The future of imaging in HF is not merely about better images but rather the ‘bigger picture’—it is about smarter, more integrated, and more personalised data that directly inform clinical decision-making. The convergence of AI, molecular imaging and advanced CMR and echocardiographic techniques is creating an unprecedented opportunity to transform HF care from a one-size-fits-all approach to a precision-guided discipline.

As Section Editor for Imaging in Heart Failure in *Current Heart Failure Reports*, I am committed to curating a collection of reviews that critically appraise these innovations, highlight translational progress, and address the practical challenges of implementation (see Table 1). I warmly invite contributions from colleagues across the imaging and heart failure communities and look forward to building a vibrant, evidence-driven discourse on how imaging can best serve our patients with HF.

**Table 1:** Key gaps in knowledge for implementation of advanced and novel imaging approaches in heart failure Topic / Section refers to the major domain covered by each section of the article. Key Knowledge Gap describes the unresolved clinical, scientific, or translational questions that the review identifies as limiting current practice. How the Section Aims to Fill It outlines the approach the imaging section of Current Heart Failure Reports intends to take in addressing each gap through commissioned reviews

Topic	Key Knowledge Gaps	Section Aims
**AI & Deep Learning in HF Imaging**	- Lack of routine clinical integration - AI algorithms lack large-scale validation for bias, generalisability, and interpretability	Reviews with clinician-focused appraisal on AI-driven automation, deep phenotyping, radiomics, and vendor-agnostic analyses for HF-specialists
**Echocardiography**	− 3D echo volumetric accuracy still approximates, compared to CMR - POCUS diagnostic accuracy in community/resource-limited settings is incompletely defined	Consensus documents on utility of advanced echocardiography and routine use of handheld (AI-enabled) POCUS
**CMR**	- Parametric mapping (T1, T2, ECV) interpretation is not fully standardised - Clinical utility of 4D flow, DTI and MR spectroscopy in HF requires further prospective evidence - CMR access for patients with implanted devices remains limited	Reviews and Meta-Analyses of emerging CMR techniques in broader HF populations with different aetiologies. Summarise existing evidence and current imaging protocols of feasibility and safety of HF patients with CIED
**Molecular & Nuclear Imaging**	- FAP-targeted PET tracers lack large-scale clinical validation - Hyperpolarized MR metabolic imaging is restricted to select centres - Transition from structural to process-level fibrosis imaging is nascent	Highlight translational progress in novel PET tracers (FAPI, FDG, MIBG), hyperpolarized MR, and their capacity to image active pathobiological processes (inflammation, fibrotic activity, metabolic remodelling)
**Cardiac Computed Tomography (CCT)**	- CT-derived strain, ECV quantification, and myocardial perfusion analyses are not yet established in routine HF work-up - The ‘one-stop-shop’ CT concept for HF needs prospective validation	Assess the expanding role of CCT—including FFR-CT, photon-counting CT, and preprocedural planning for structural interventions—in the comprehensive evaluation of HF patients
**Digital Twins & Computational Modelling**	- Digital twins remain largely in the research domain and clinical translation lacks regulatory frameworks - No in-silico clinical trial standards exist	Explore the potential of patient-specific computational models to predict therapy response in silico and outline the translational roadmap toward clinical and regulatory readiness
**Multimodality Integration & Precision Phenotyping**	- Multi-modality derived imaging data are scarce - No consensus on how multimodal data should inform individualised treatment decisions in HF	Advocate for convergence of AI, molecular imaging, and advanced echo/CMR techniques into integrated, predictive, patient-specific frameworks that move HF care toward precision medicine
**Equity**,** Training & Cost-Effectiveness**	- Advanced imaging innovations are concentrated in well-resourced academic centres - Cost-effectiveness of novel strategies is unproven - Physician training does not cover AI, computational imaging, and multimodality integration	Address practical implementation challenges—equitable dissemination (handheld ultrasound, cloud platforms), evidence for cost-effectiveness, and curricular evolution for the next generation of HF specialists

*3D* three-dimensional, *4D* four-dimensional, *AI* artificial intelligence, *ATTR-CM* transthyretin amyloidosis cardiomyopathy, *CCT* cardiac computed tomography, *CMR* cardiovascular magnetic resonance, *CRT* cardiac resynchronisation therapy, *CT* computed tomography, *DTI* diffusion tensor imaging, *ECV* extracellular volume, *EHR* electronic health record, *FAP* fibroblast activation protein, *FAPI* fibroblast activation protein inhibitor, *FDG* fluorodeoxyglucose, *FFR-CT* CT-derived fractional flow reserve,* HF* heart failure, *LVAD* left ventricular assist device, *MIBG* metaiodobenzylguanidine, *MR* magnetic resonance, *MRS* magnetic resonance spectroscopy, *PCCT* photon-counting computed tomography, *PET* positron emission tomography, *POCUS* point-of-care ultrasound, *STE* speckle-tracking echocardiography

## Data Availability

No datasets were generated or analysed during the current study.
